# Tip of the iceberg: Extra–haematological consequences of early iron deficiency

**DOI:** 10.7189/jogh.05.020304

**Published:** 2015-12

**Authors:** Fareeda Sohrabi

**Affiliations:** Queen Mary University of London, Barts and the London School of Medicine and Dentistry, London, UK

Over a third of the global population is estimated to have Iron Deficiency (ID). It manifests during all stages of life and especially during times of accelerated growth phases, mainly affecting infants, adolescents and pregnant women.

ID has been shown to be associated with poorer cognitive, motor and behavioural development, including lower intelligence quotients (IQ) in children [1]. It is also associated with poorer pregnancy outcomes for mothers and infants. This raises the question of the global effects of ID, in terms of cognitive abilities, economics and productivity. This viewpoint reviews some of the recently discovered extra–haematological effects of ID and its relevance to modern–day society, in both developed and developing countries.

## DEFINING IRON DEFICIENCY

**Figure 1:**
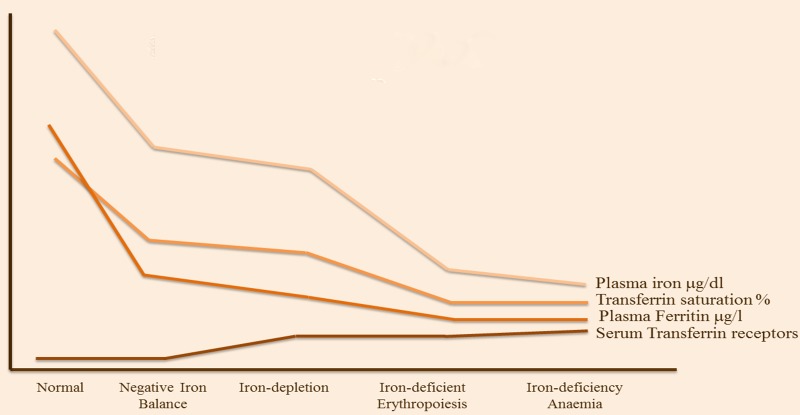
Serum iron changes in .the stages of iron deficiency. Source of data: Clark, 2008 [2].

Long–term negative iron balance leaves the body with no measurable iron stores, causing insufficient supplies for cellular processes. ID is at the end of a continuum (**Figure 1**), where the most severe clinical consequence is iron deficiency anaemia (IDA). The other forms of ID tend to be asymptomatic. A good indicator of ID is by diagnosis of anaemia in combination with low serum ferritin, as established by the World Health Organisation (WHO). It is estimated that ID is twice as prevalent as anaemia and that approximately half of all anaemias are due to ID. Approximately one quarter of the world’s population has anaemia, with the most common cause being due to ID. In the UK, 21% of female adolescents and 18% of women aged 16–64 years are iron–deficient [3].

## CONSEQUENCES OF IRON DEFICIENCY IN PREGNANCY

Pregnancy is a physiologically iron–depleting state as maternal iron stores preferentially transfer to the foetus *in utero*. Many women enter the pregnancy state with anaemia, as previous studies have shown, and many having no iron stores when becoming pregnant [4]. This suggests that they have insufficient levels required for adequate development of the foetus and placenta and for blood loss during childbirth.

Maternal haemoglobin levels have been found to have a U–shaped association with low birth weight infants [5,6]. An Indian retrospective study showed a negative correlation between serum ferritin <10 μg/L and birth weight [4]. Many interventional studies have shown increases in birth weight by a modest amount when pregnant women are given iron–repletion [7].

## CONSEQUENCES OF IRON DEFICIENCY IN CHILDREN

Although rates of IDA have been steadily declining for decades, it is markedly prevalent in poor, minority and immigrant groups [8,9]. A recent study looking at specific cognitive domains of ID infants, found that they were suffering from poorer attention and memory processing (lower scores on recognition memory, object permanence and memory encoding/retrieval) [9]. Another study showed that infants with IDA had poorer high–speed information processing times, in comparison to non–anaemic infants– a predictor of future IQ [1].

Rodent models have shown that during critical brain development, a lack of iron causes defects in post–translational incorporation of iron into haemoproteins (haemoglobin and cytochromes) [8]. The lack of cytochromes in the frontal lobe and hippocampus suggests a reduction in cerebral metabolism due to low levels of ATP [8]. Iron–containing monoamines are predictably acutely affected but it is postulated to cause longer–term effects on dopamine metabolism in particular, which is known to regulate cognition and emotion [8].

Neonatal serum ferritin levels which correlate with low brain iron and neurodevelopmental abnormalities is approximately <35 μg/L [10]. There is also evidence to suggest that after a critical period of ID in the brain of rats, iron–repletion thereafter will not reverse the structural deficits [8].

Interestingly in the Costa Rican study of infants aged five years [11], whose iron status’ had been corrected from infancy, still scored lower on mental and motor functioning tests, consistent with other studies – even in the case of corrected haemoglobin levels (>100 g/L).

Studies assessing the effects of ID on social behaviour found that iron–deficient children were more wary, hesitant, unhappy and kept closer to their mothers [8]. In another study, infants who did not receive prophylactic iron also showed poorer social–emotional outcomes [12].

## CONSEQUENCES OF IRON DEFICIENCY IN ADULTS: EFFECTS ON PRODUCTIVITY

The effects of IDA can be insidious and subtle. In an individual with ID in infancy or in early childhood, the irreversible effects on cognition and social development are likely to have an impact in later life. Consequently, the effect of ID on adult productivity, which has been shown to affect the economy in the long run, is not well recognised, and therefore significant consideration in tackling the problem is lacking [12].

Chronic undetected ID from childhood may lead to lower than expected IQs and fewer skill acquisitions in adults affecting their productivity levels. One of the most tangible outcomes of working adults is of productivity measures. It can be used to objectively compare outcomes between countries. In this context, productivity can be defined as the efficiency of adults in the workplace, with Gross Domestic Product (GDP) used as the unifying unit to compare the effects on adults with ID to those with normal iron status. Although most data are mathematically derived due to variations in data collection in less developed countries, this is still important in understanding some of the long–term effects of ID.

**Figure:**
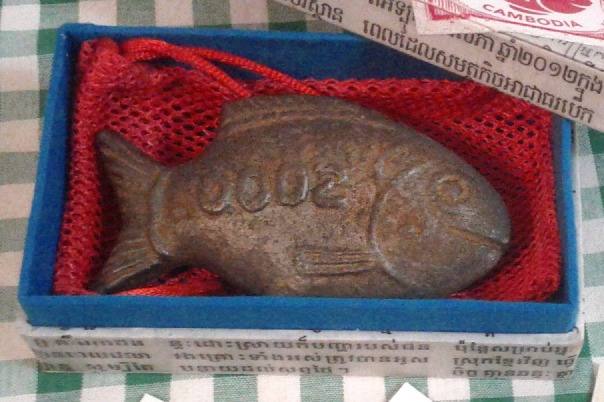
Photo: A Cambodian Lucky Iron Fish, which is added to cooking water to reduce iron deficiency. Photo by Dflock (Own work) [CC BY-SA 4.0], via Wikimedia Commons

Losses in productivity may be attributable to reduced aerobic capacity both acutely or from persisting suboptimal academic/emotional/social performance from infancy [13]. A study carried out in low–income Asia estimated productivity losses of 17% for workers in labour–intensive jobs, and 5% for moderately active jobs due to ID [12].

An economic study of ten countries assessing the impact of ID on GDP showed a loss of 2.0–7.9% [13] (estimated US$ 9.78 billion/annum, using 2005 figures [14]). The effects of ID on an individual can be relative to the development status of the country they are living in which also has implications for the relative accountability of GDP losses in any given individual.

In a less developed country, the majority of workers are in labour–intensive jobs and the prevalence of ID is likely over 50%. ID is less likely to have an impact on GDP losses but may have a larger impact on their lives in supporting their family/community. On the other hand, in developed countries, where most working adults are in white–collar jobs and the prevalence of ID is considerably lower, they are still likely to have a larger impact on GDP.

## PUBLIC HEALTH CHALLENGES

The insidious and subclinical effects of ID, regardless of when it appears in an individual’s life time, are clearly problematic. The difficulty with analysing the extra–haematological consequences of ID is mainly due to the fact that there are a multitude of factors which make it very difficult to separate the true effects of chronic negative iron balance.

The evidence for the associations between maternal ID and its effect on pregnancy outcomes is based on some important studies. Anaemia in the first and second trimester of pregnancy can demonstrate warning signs of subclinical ID, putting unborn infants in a sub–optimal environment for brain development.

Consistent results highlight the suboptimal development of the hippocampus, which is a seminal region of the brain used in memory function. The most surprising and worrying finding in this area was the fact that iron–repletion at any stage after the first two years of life has minimal effect on the reversibility of neurodevelopment in the hippocampus (although it may improve in other domains) [8].

The evidence regarding the predictability of poorer memory and learning in children with early ID is concordant with most papers studying cognition in children. Some of the specific differences observed included poorer attention, memory processing and lower IQ scores [8].

There is consistency of study results showing modest improvement in function with iron–repletion therapy. With pregnant women, iron–repletion tends to increase the birth weight of the infant when compared to predicted weight, but there is still a lack of normalisation. Similarly, iron–repletion in young children tends to improve cognitive and behavioural parameters, but the differences are not normalised. One of the biggest limitations to these findings is the fact that trials have not looked at reversibility of function long–term, so it is unclear whether iron–repletion eventually causes normalisation of cognitive abilities or not.

It is well understood that the loss of aerobic capacity in IDA causes acute changes in productivity. The effects of chronic negative iron balance have been implicated in financial losses made by whole nations. The study by Horton and Ross [15] demonstrates a crude but humbling awareness of the staggering loss of economy in the working population, and it highlights some of the problems in this area in tangible terms. Although the dominating employment sectors vary from country to country, it is still an appreciable loss made globally.

## References

[1] Lozoff B, De Andraca I, Castillo M (2003). Behavioral and developmental effects of preventing iron–deficiency anemia in healthy full–term infants.. Pediatrics.

[2] Clark SF (2008). Iron deficiency anemia.. Nutr Clin Pract.

[3] Heath AL, Fairweather–Tait SJ (2002). Clinical implications of changes in the modern diet: iron intake, absorption and status.. Best Pract Res Clin Haematol.

[4] Singla PN, Tyagi M, Kumar A (1997). Fetal growth in maternal anaemia.. J Trop Pediatr.

[5] Murphy JF, O'Riordan J, Newcombe RG (1986). Relation of haemoglobin levels in first and second trimesters to outcome of pregnancy.. Lancet.

[6] Menendez C (1994). Vitamin A and iron supplementation in pregnancy.. Lancet.

[7] Lozoff B, Georgieff MK (2006). Iron deficiency and brain development.. Semin Pediatr Neurol.

[8] Carter RC, Jacobson JL, Burden MJ (2010). Iron deficiency anemia and cognitive function in infancy.. Pediatrics.

[9] Wigglesworth J, Baum H. Iron dependent enzymes in the brain. In: Youdim M, ed. Brain iron: Neurochemical and behavioural aspects. New York: Taylor and Francis, 1988.

[10] Dallman PR, Siimes MA, Manies EC (1975). Brain iron: persistent deficiency following short–term iron deprivation in the young rat.. Br J Haematol.

[11] Lozoff B, Jimenez E, Wolf AW (1991). Long–term developmental outcome of infants with iron deficiency.. N Engl J Med.

[12] Williams J, Wolff A, Daly A, MacDonald A, Aukett A, Booth IW (1999). Iron supplemented formula milk related to reduction in psychomotor decline in infants from inner city areas: randomised study.. BMJ.

[13] Hunt JM (2002). Reversing productivity losses from iron deficiency: the economic case.. J Nutr.

[14] The World Bank. GDP (current US$). Secondary GDP (current US$). 2015. Available: http://data.worldbank.org/indicator/NY.GDP.MKTP.CD. Accessed: 5 January 2015.

[15] Horton S, Ross J (2003). The Economics of Iron Deficiency.. Food Policy.

